# Inflammatory masquerade features in adult-onset *CLN*-associated retinal degeneration in a multiethnic Asian cohort

**DOI:** 10.1186/s12348-026-00623-9

**Published:** 2026-07-28

**Authors:** Srinivasan Sanjay, Soon-Phaik Chee, Rachael W. C. Tang, Weng Khong Lim, Yasmin Bylstra, Tien-En Tan, Ranjana Mathur, Choi Mun Chan, Saadia Farooqui, Beau J. Fenner

**Affiliations:** 1 Department of Cataract and Comprehensive Ophthalmology, Singapore National Eye Centre, 11 Third Hospital Avenue, Singapore, 168751 Singapore; 2 Ophthalmology & Visual Sciences Academic Clinical Programme, Duke-NUS Medical School, SGH Campus 8 College Rd, Singapore, 169857 Singapore; 3 Department of Ocular Immunology and Inflammation, Singapore National Eye Centre, 11 Third Hospital Avenue, Singapore, 168751 Singapore; 4 Singapore Eye Research Institute, Singapore, The Academia, 20 College Rd, Level 6 Discovery Tower, Singapore, 169856 Singapore; 5 National University of Singapore, Department of Ophthalmology, Yong Loo Lin School of Medicine1E Kent Ridge Road, NUHS Tower Block, Level 7, Singapore, 119228 Singapore; 6 Ocular Genetics Service, Singapore National Eye Centre, 11 Third Hospital Avenue, Singapore, 168751 Singapore; 7 Laboratory of Genome Variation Analytics, Genome Institute of Singapore, Agency for Science, Technology and Research, Biopolis Way, One North (JTC), 60 Biopolis St, Singapore, 138672 Singapore; 8 SingHealth Duke-NUS Institute of Precision Medicine (PRISM) Level 9, National Heart Centre, 5 Hospital Drive, Singapore, 169609 Singapore; 9 Department of Surgical Retina, Singapore National Eye Centre, 11 Third Hospital Avenue, Singapore, 168751 Singapore; 10 Nuffield Laboratory of Ophthalmology, Nuffield Department of Clinical Neurosciences, University of Oxford, Oxford, United Kingdom Level 6, West Wing, John Radcliffe Hospital, Headington, Oxford, OX3 9DU UK; 11 Oxford Eye Hospital, West Wing of the John Radcliffe Hospital, Level LG1, Headley Way, Headington, Oxford University Hospitals NHS Foundation Trust, Oxford, OX3 9DU United Kingdom; 12 Department of Medical Retina and Ocular Genetics, Singapore National Eye Centre, 11 Third Hospital Avenue, Singapore, 168751 Singapore; 13 Department of Paediatric Ophthalmology, Singapore National Eye Centre, 11 Third Hospital Avenue, Singapore, 168751 Singapore

**Keywords:** Neuronal ceroid lipofuscinosis, Masquerade syndrome, Ocular inflammation, Uveitis

## Abstract

**Purpose:**

To describe the clinical, imaging, and genetic features of non-syndromic neuronal ceroid lipofuscinosis (NCL)-associated retinal degeneration in a multi-ethnic Asian cohort, with particular emphasis on cases that were initially considered in the differential diagnosis of non-neoplastic uveitis masquerade syndromes.

**Methods:**

This retrospective case series reviewed seven unrelated Singaporean patients (Chinese and Indian ancestry) with genetically confirmed CLN-associated inherited retinal degeneration. Clinical presentation, inflammatory features, longitudinal multimodal imaging, electrophysiology, and molecular findings were analyzed.

**Results:**

Symptom onset ranged from 18 to 54 years. Presenting symptoms included “blurred vision/floaters (n = 3)”, “nyctalopia (n = 3)”, and “hemeralopia (n = 1)”. Five patients presented with active ocular inflammation, such as anterior chamber and vitreous cells and retinal vasculitis. Notably, the other two patients (harboring *CLN2* and *CLN3* variants) did not show overt anterior or posterior segment inflammation; however, their striking structural and imaging similarities to inflammatory retinopathies prompted their inclusion in the masquerade differential. Across all seven patients, serial optical coherence tomography (OCT) revealed relentless outer retinal atrophy and loss of the ellipsoid zone. One of the five patients received corticosteroid and immunosuppressive therapy but showed only a limited response, whereas the remaining four patients responded favourably to topical, periocular, or oral corticosteroids. During a follow-up of 2.7–33.1 years, median best-corrected visual acuity declined to logMAR 1.90. Systemic health remained stable without neurological symptoms, except for one *CLN2-*associated case developing adult-onset neurosensory hearing loss. Pathogenic variants in *CLN1*, *CLN2*, *CLN3*, *CLN5*, and *CLN7* were identified through genetic testing. CLN-associated disease accounted for 1.1% of the inherited retinal disease families in the cohort.

**Conclusions:**

Retinal degeneration associated with *CLN1*, *CLN5*, and *CLN7* variants frequently presented with intraocular inflammation (ranging from anterior to panuveitis) in our cohort. Yet even patients without obvious inflammatory signs experienced a relentless retinal decline that closely mimicked the structural damage seen in severe posterior uveitis. These findings highlight why early genetic testing is so crucial when working up atypical, symmetrical posterior uveitis or suspected masquerade syndromes.

## Introduction

Uveitis masquerade syndromes are conditions that mimic intraocular inflammation but arise from non-inflammatory or non-infectious pathology [1]. They account for approximately 5% of cases seen in uveitis clinics and pose a significant diagnostic challenge, often leading to delayed diagnosis and inappropriate immunosuppressive therapy [2–4]. While neoplastic masquerades are well recognised, non-neoplastic causes are increasingly appreciated and include a spectrum of inherited retinal diseases (IRDs), which may present with mild anterior chamber or vitreous cells and retinal changes resembling posterior uveitis.

Among non-neoplastic masquerade syndromes, IRDs represent an important and under-diagnosed subgroup [5]. Previous cohort studies have shown that IRDs account for up to one-third of non-neoplastic inflammatory masquerades and are associated with the longest diagnostic delays, often exceeding several months [6]. Symmetry of disease, poor response to immunosuppression, and progressive outer retinal atrophy are key clinical clues. However, overlap with inflammatory phenotypes frequently leads to prolonged treatment courses before the correct diagnosis is established.

Ceroid lipofuscinosis neuronal (CLN) genes encode 13 proteins that localize throughout the endomembrane system to regulate a variety of cellular processes. Mutations in CLN genes cause neuronal ceroid lipofuscinoses (NCLs) which are a heterogeneous group of lysosomal storage disorders characterised by the accumulation of auto fluorescent lipopigment in neurons and other tissues [7, 8]. Classic NCL phenotypes present in childhood with progressive neurodegeneration, epilepsy, cognitive decline, and premature death, with retinal degeneration often representing an early manifestation [8]. 

To date, more than 13 *CLN* genes have been identified, each associated with variable age of onset and clinical severity [9, 10]. 

In contrast to the well-described paediatric syndromic forms, adult-onset and non-syndromic *CLN*-associated retinal degeneration is increasingly recognised [11]. These patients may present initially with isolated visual symptoms, lack overt neurological features for many years, and exhibit retinal phenotypes resembling retinitis pigmentosa or cone–rod dystrophy. Importantly, inflammatory signs such as vitreous cells, retinal vasculitis, cystoid macular edema [CME], or optic disc hyperemia have been reported, further compounding diagnostic uncertainty and leading to misclassification as posterior uveitis or autoimmune retinopathy.

Reports describing *CLN*-associated retinal degeneration presenting as an inflammatory masquerade remain sparse, particularly in Asian populations. The prevalence, clinical spectrum, and genetic distribution of such cases in Southeast Asia remain unknown, despite growing access to genetic testing and multimodal retinal imaging [12, 13]. Improved recognition of IRD is essential to avoid unnecessary long-term immunosuppression, reduce diagnostic delay, and enable appropriate genetic counselling and surveillance for potential systemic involvement.

In the current study, we outline the clinical, multimodal imaging, and genetic profiles of a multi-ethnic Asian cohort with CLN-associated retinal degeneration who had non-neoplastic uveitis masquerade syndromes. To fully capture the diagnostic challenge, we deliberately included both patients without obvious intraocular inflammation and those whose presentation mimicked inflammatory disease purely based on structural imaging and clinical progression. We also detail a representative case that endured prolonged, ineffective immunosuppression before a genetic diagnosis was reached, and we provide context on the overall prevalence of CLN-driven disease within a large IRD registry at a tertiary centre.

## Methods

### Patient recruitment and clinical evaluation

Unrelated probands with IRDs, together with their affected relatives, were recruited from a tertiary ophthalmic centre in Singapore. Patients were enrolled into the study between March 2021 and March 2025. Retrospective chart data were reviewed, extending to the patients’ initial presentations at the study centre, which may have preceded their enrolment. Comprehensive ophthalmic examination was performed for all participants, including measurement of best-corrected visual acuity (BCVA), multimodal retinal imaging which included color fundus photos, autofluorescence, spectral domain optical coherence tomography (OCT), fundus fluorescein angiography (1 patient), electroretinogram (ERG) and visual field testing.

### Genetic testing and bioinformatic analysis

Genetic evaluation was conducted using either whole-exome sequencing or targeted IRD-focused gene panel testing. Identified variants were further analyzed through in silico prediction tools to assess pathogenicity, conservation, and potential functional impact. Targeted IRD panel testing was performed in patients IRD0208, IRD0119, IRD0346, and IRD0350, while whole-exome sequencing was performed in patients IRD0827, IRD0499, and IRD0454 [14, 15]. The study was approved by the SingHealth Institutional Review Boards and carried out in accordance with the ethical standards of the 1964 Declaration of Helsinki and its later amendments. Informed consent was obtained from all participants.

## Results

### Cohort characteristics

Seven unrelated Singaporean patients of Chinese (4) and Indian (3) ancestry with CLN-associated retinal degeneration were identified. Age at symptom onset ranged from 18 to 50 years (median 29 years), and age at presentation from 26 to 55 years. There were 4 males and 3 females in this series. Table 1 illustrates the clinical and genetic findings of our cohort. Presenting symptoms were bilateral, asymmetric at presentation and included blurred vision or visual floaters in three patients, nyctalopia in three patients, and hemeralopia in one patient. None of the patients had neurological symptoms. Extensive infectious and autoimmune work-up was specifically documented for the illustrative case IRD0827 before immunosuppressive treatment, whereas this level of standardized work-up was not uniformly available in complete detail for every patient because of the retrospective design and multi-source referral pathway.

**Table 1:** Clinical and genetic findings in patients with CLN-associated retinal degeneration

Patient	Ethnicity	Phenotype	Age at symptom onset and presentation	Initial BCVA logMAR (OD|OS)	Genotype	Time at onset of Inflammation	Diagnosis	Treatment	Final BCVA logMAR (OD|OS)	Follow up duration (years)
IRD0827	F / C	RCD	25 / 25.5	0.40 | 0.30	*CLN1*(*PPT1*) c.272 A > C / c.491G > A	Presentation	Panuveitis	Topical/oral/IMT/Biologics	0.30 | 0.18	2.7
IRD0499	M / C	RCD + hearing loss	49 / 50.2	0.00 | 0.10	*CLN2*(*TPP1*) c.604 C > T / c.589 C > T	—	—	—	0.18 | 0.10	5.3
IRD0454	M / C	RCD	18 / 54.7	0.18 | 0.18	*CLN3* c.837 + 5G > A / c.534–2 A > G	—	—	—	1.90 | 2.30	12.8
IRD0208	M / C	CRD	32 / 32.3	1.30 | 0.40	*CLN5* c.1009 C > G hom.	Course	IU	Topical/Periocular	1.90 | 1.90	11.9
IRD0119	F / I	RCD	50 / 51.4	0.78 | 1.00	*CLN7*(*MFSD8*) c.1361T > C hom.	Course	AU + IU	Topical/Periocular/oral	2.70 | 2.30	33.1
IRD0346	M / I	RCD	29 / 29.5	0.60 | 0.70	*CLN7*(*MFSD8*) c.1361T > C hom.	Course	AU + IU	Topical/Periocular/oral	3.00 | 2.30	24.4
IRD0350	F / I	RCD	20 / 43.8	0.00 | 0.00	*CLN7*(*MFSD8*) c.1361T > C hom.	Course	AU + IU	Topical/Periocular	0.10 | 0.10	4.9

M- Male, F- Female, C- Chinese, I-Indian, IRD- Inherited retinal disease, RCD- Rod cone dystrophy, CRD- Cone-rod dystrophy, BCVA- Best corrected visual acuity, OD- Right eye, OS- Left eye, IU- Intermediate uveitis, AU- Anterior uveitis

Longitudinal visual outcomes were variable across the cohort. Some patients experienced relatively rapid decline in visual acuity, particularly during mid-to-late adulthood, whereas others showed a more gradual course or remained visually stable over the follow-up period. Estimated rates of BCVA deterioration ranged from negligible change to approximately 0.21 logMAR/year, highlighting the marked inter-individual variability in disease progression. Given the small sample size and genetic heterogeneity of the cohort, no meaningful genotype–phenotype correlations could be established. Follow-up duration ranged from 2.7 to 33.1 years (median 11.9 years).

### Clinical and imaging features

Although all patients had bilateral retinal disease that was largely symmetrical overall, three showed asymmetry at presentation, with one eye more severely affected than the other. Four of the seven patients (IRD0119, IRD0208, IRD0346, and IRD0350) developed overt inflammatory signs at some stage of their clinical course, including anterior chamber inflammation (0.5 + to 3 + cells by SUN criteria) and vitritis. Another patient (IRD0827) initially presented with non-occlusive retinal venular sheathing, hyperaemic optic discs, and CME, raising suspicion of an inflammatory retinal disorder before subsequently being diagnosed with CLN1-associated retinal degeneration. In contrast, the remaining two patients (IRD0499 and IRD0454), with CLN2- and CLN3-associated disease respectively, did not demonstrate definite active inflammation. However, their progressive retinal degeneration, characterised by widespread pigmentary changes, vascular attenuation, and optic disc pallor, closely resembled the sequelae of previously active or “burnt-out” uveitis, leading them to be considered within the differential diagnosis of uveitic masquerade syndromes. Table 1 shows the clinical characteristics of the cohort.

**Fig. 1:**
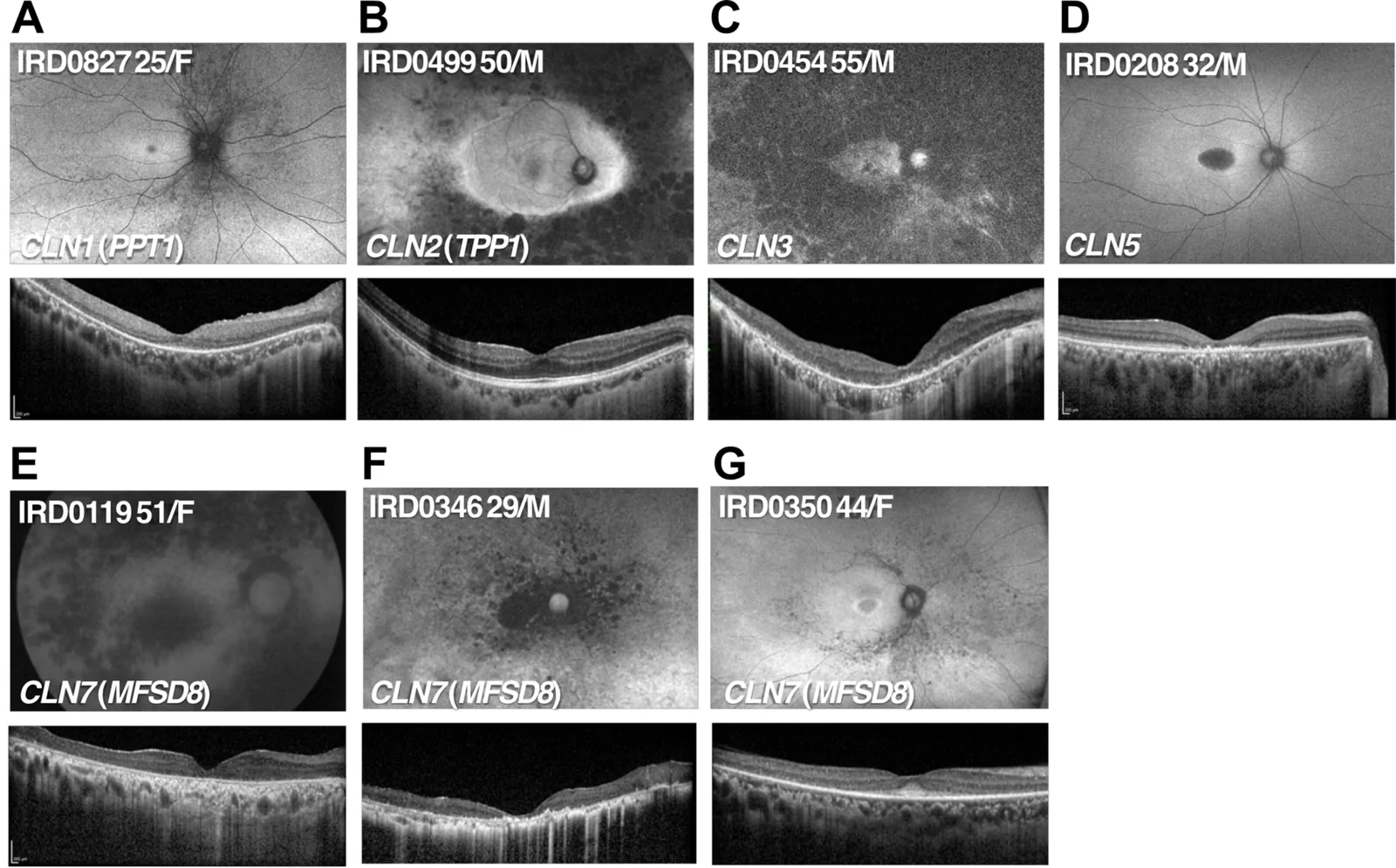
Spectrum of autofluorescence and optical coherence tomography findings in patients with CLN-associated retinal degeneration. Representative fundus autofluorescence images from different patients in the cohort illustrate heterogeneous patterns of retinal involvement, including diffuse, peripapillary, and nummular hypoautofluorescence. Corresponding spectral-domain OCT images demonstrate variable degrees of outer retinal attenuation, ellipsoid zone loss, and retinal thinning, reflecting the phenotypic diversity of CLN-associated retinal degeneration at a cross-sectional time point. Most cases demonstrated rod-cone dystrophy-like phenotypes, whereas IRD0208 (CLN5-associated disease) demonstrated a predominantly cone-rod dystrophy-like phenotype with ERG showing global cone-rod dysfunction

Fundus autofluorescence imaging revealed variable patterns of retinal degeneration including generalized disease, predominantly peripapillary disease, and nummular hypo-autofluorescence with varying sizes from coin to more than a disc dioptre, across the cohort. (Fig. 1) OCT demonstrated progressive outer retinal attenuation with ellipsoid zone loss in all patients, with cystoid spaces present in 4 patients at time of presentation to ocular inflammation services. Visual fields (Humphreys’ and Goldmann) showed progressive decline in all the patients over the course of the follow up. Electrophysiology was done for all the patients during their follow up. The full-field ERG scotopic/mixed and photopic responses were attenuated and finally undetectable which indicated a marked rod-cone dysfunction. The multifocal ERG was also markedly attenuated. Fundus fluorescein angiography was done in only one patient (IRD0827) and will be described in the following paragraphs.

Four patients had an established diagnosis of IRD before developing ocular symptoms suggestive of intraocular inflammation, including eye pain, redness, blurred vision, and floaters. Among them, IRD0208 presented with bilateral floaters accompanied by predominantly anterior vitritis, mild vitreous haze, and CME. Based on the clinical impression of an inflammatory process, the patient was treated with a tapering course of topical prednisolone acetate 1% for four weeks together with bilateral periocular triamcinolone acetate injections (40 mg).

The remaining three patients (IRD0119, IRD0346, and IRD0350) were managed similarly with topical prednisolone acetate 1% tapered over six weeks and bilateral periocular triamcinolone acetate injections. In two patients (IRD0346 and IRD0350), mild inflammation persisted despite periocular steroid treatment, prompting the addition of a short course of oral prednisolone approximately three weeks later. Oral prednisolone was started at 30 mg daily and tapered over four weeks. Treatment details and clinical responses were obtained through retrospective review of the medical records.

The treatment of patient with pan uveitis (IRD0827) will be explained in the following paragraphs.

At final follow-up of this cohort, BCVA ranged from logMAR 0.10–3.00 (median 1.90) in the right eye and logMAR 0.10–2.30 (median 1.90) in the left eye, reflecting substantial visual decline in most patients.

### Illustrative case (Patient IRD0827)

Patient (IRD0827) (Table 1) initially presented at another centre with bilateral blurred vision and floaters. Examination revealed anterior chamber cells (2+, SUN Classification) anterior vitreous cells, optic disc swelling, and CME in both eyes. Ellipsoid zone (EZ) was intact at this presentation. An extensive infectious (including syphilis antibodies, QuantiFERON Gold TB) and autoimmune evaluation was unremarkable. The patient was diagnosed with idiopathic intermediate uveitis.

The patient underwent sequential treatment at that centre with topical and systemic corticosteroids, azathioprine, adalimumab, and methotrexate over couple of years, with only transient or minimal improvement in symptoms and persistent structural abnormalities. Serial OCT images available with the patient showed loss of EZ and outer retinal atrophy. Patient decided to seek a second opinion.

**Fig. 2:**
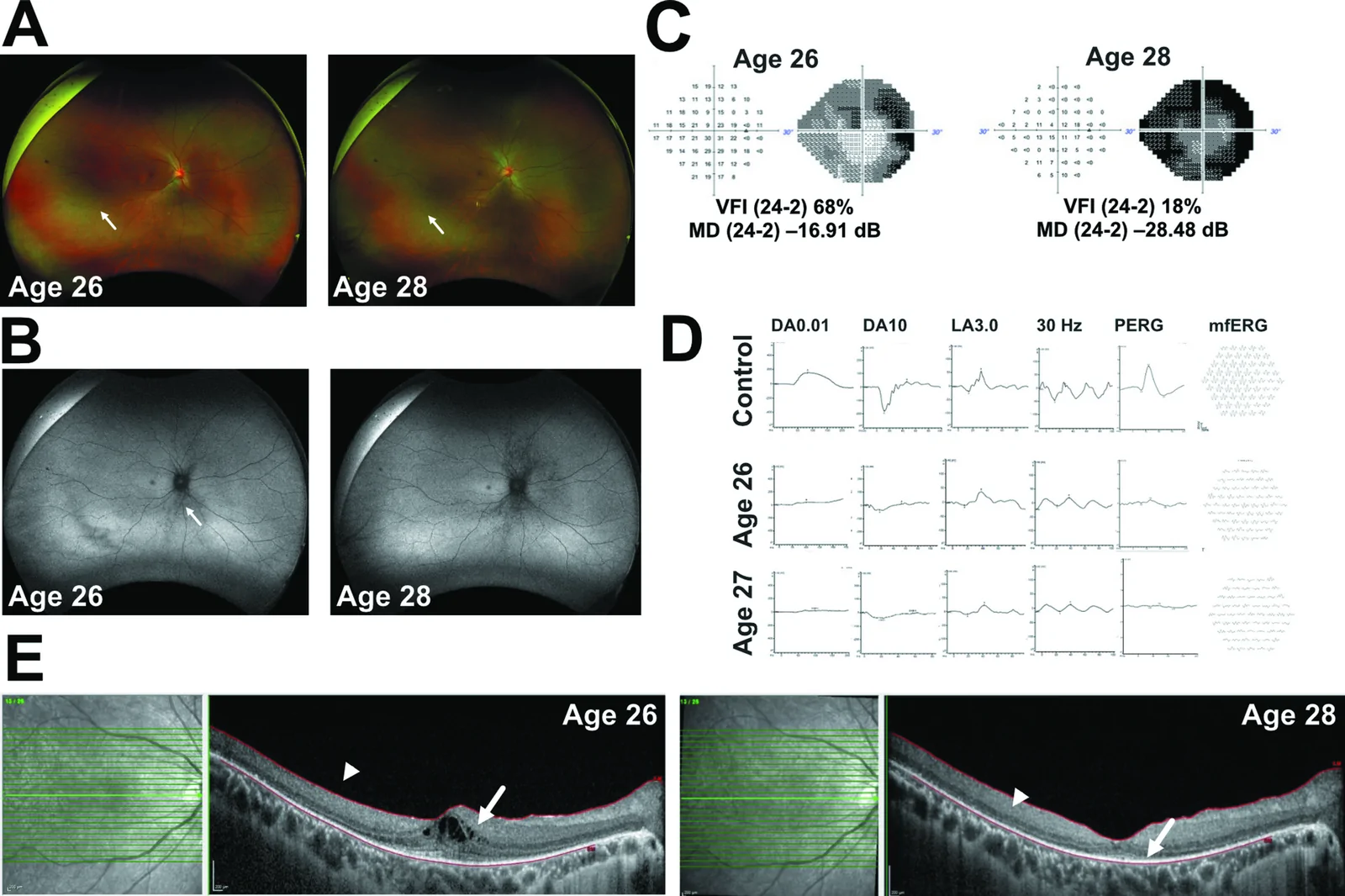
Multimodal imaging demonstrates progressive CLN1-associated retinal degeneration masquerading as posterior uveitis in Patient IRD0827. (**A**) Ultra-widefield colour fundus photographs (Optos) of the right eye at age 26 and age 28 demonstrate progressive diffuse retinal pigmentary disturbance with increasing optic disc pallor and vascular attenuation. The arrow highlights an area of evolving mid-peripheral retinal atrophy. (**B**) Fundus autofluorescence images of the right eye at age 26 and age 28 years show progressive enlargement of peripapillary and diffuse hypoautofluorescence, consistent with advancing retinal pigment epithelium and outer retinal loss. (**C**) Humphrey visual field testing (24 − 2) demonstrates marked interval deterioration over two years, with worsening grayscale defects, reduced visual field index (VFI), and increased mean deviation (MD), reflecting progressive generalized visual field constriction. (**D**) Full-field and macular electrophysiology compared with age-matched control. Dark-adapted (DA 0.01 and DA 10.0), light-adapted (LA 3.0), 30-Hz flicker, pattern electroretinography (PERG), and multifocal ERG (mfERG) recordings show severely attenuated and delayed responses at age 26 and age 27 years, indicating profound generalized rod–cone dysfunction with macular involvement. (**E**) Spectral-domain optical coherence tomography (OCT) through the fovea at age 26 demonstrates cystoid macular edema, fragmented ellipsoid zone (arrow), and inner retinal granularity (arrowhead). At age 28 years, following topical dorzolamide therapy, intraretinal cystoid changes have resolved; however, there is progressive loss of outer retinal layers, including the ellipsoid zone, consistent with ongoing retinal degeneration

**Fig. 3:**
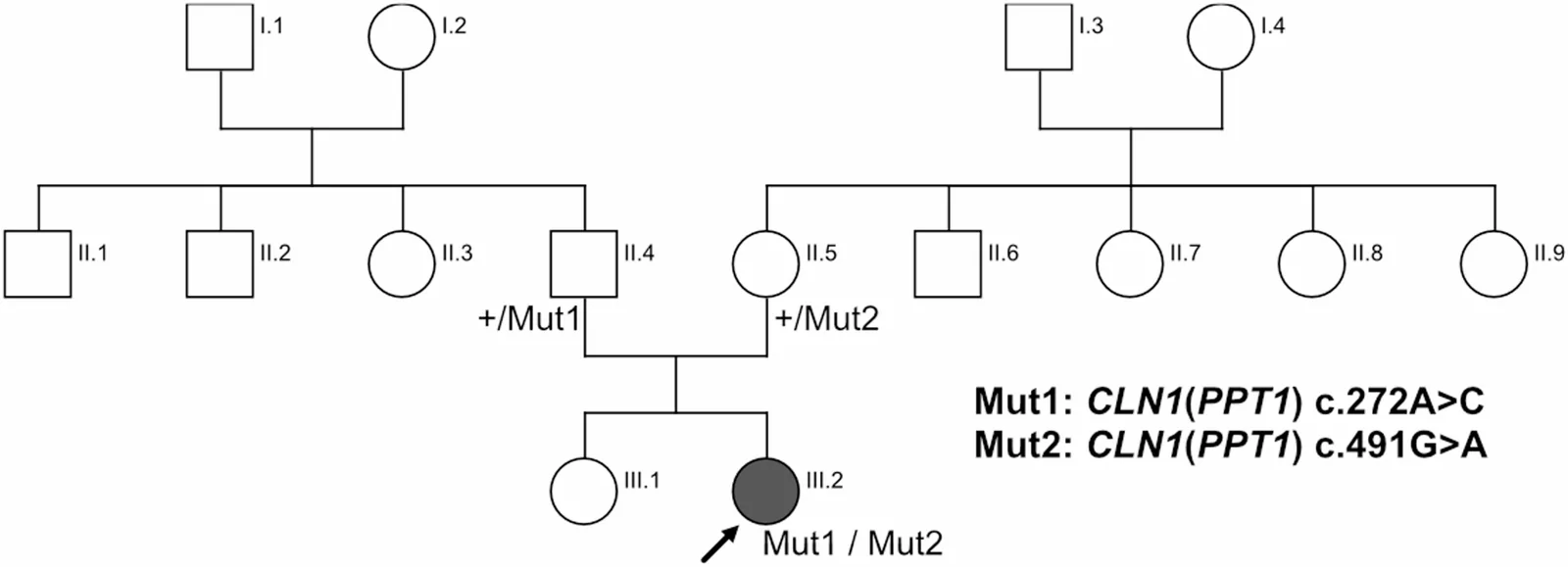
Pedigree of Patient IRD0827 with CLN1-associated retinal degeneration. Pedigree demonstrating autosomal recessive inheritance. The proband is indicated by the filled symbol. The father had a history of colour vision deficiency, while the proband’s sibling is clinically unaffected. Parental testing confirmed compound heterozygous CLN1 (PPT1) variants in trans in the proband

On presentation to our ocular immunology and inflammation services, she still had floaters and blurring of vision in both eyes. She had stopped all medications prescribed from her previous centre for about 3 months. BCVA was right (OD) 20/50 (logMAR 0.40) and left (OS) 20/40 (logMAR 0.30), with vitreous cells, disc hyperaemia and cystoid changes in the macula with constricted visual fields bilaterally. Multimodal imaging (Fig. 2) demonstrated progressive pigmentary retinal degeneration, no bony spicules, worsening peripapillary hypo-autofluorescence, concentric visual field constriction, and severe generalized rod and cone dysfunction on ERG. Macular OCT showed cystoid changes, inner retinal granularity, ellipsoid zone and progressive outer retinal loss. Ongoing disease progression and poor response to immunosuppression prompted reconsideration of the diagnosis to auto immune retinopathy. She was restarted on adalimumab 40 mg two weekly and with no further improvement over 4 months was administered weekly for 3 months. She then underwent genetic testing for IRD with our ocular genetic services and was diagnosed with CLN1- associated retinal degeneration. The pedigree chart is shown in Fig. 3. Table 2 shows the genotyping outcomes for the patient.

**Table 2:** Genotyping outcomes for patient with *CLN1* (*PPT1*)-associated retinal degeneration and parents

	Variant	ACMG classification
Proband (IRD0827)		
	*CLN1 (PPT1)* c.272 A > C (p.Gln91Pro) het.	Pathogenic
	*CLN1 (PPT1)* c.491G > A (p.Arg164Gln) het.	Likely pathogenic
Mother of IRD0827		
	*CLN1 (PPT1)* c.491G > A (p.Arg164Gln) het.	Likely pathogenic
Father of IRD0827		
	*CLN1 (PPT1)* c.272 A > C (p.Gln91Pro) het.	Pathogenic

Once the diagnosis of CLN1- associated retinal degeneration was established, immunosuppressive therapy was tapered over 5 months by increasing the frequency of administration, to two weeks and subsequently to 3 weeks after a couple of months and then finally to a monthly regimen, The immunosuppressive medication was completely stopped after 5 months. Cystoid changes were managed with topical dorzolamide 2% thrice daily for both eyes, resulting in resolution of intra-retinal fluid but progressive outer retinal degeneration. After three years of follow-up, her BCVA was OD 20/40 (logMAR 0.30) and OS 20/30 (logMAR 0.18) with a small central island of retained outer nuclear layer and ellipsoid band on OCT. The patient is on long term topical dorzolamide with no apparent ocular side effects. The patient remained systemically well, with no neurological abnormalities identified on clinical evaluation.

### Genetic findings

Among 650 families with IRD evaluated within the institutional IRD registry between March 2021 and March 2025, seven affected individuals from seven different families. (1.1%) were identified with *CLN*-associated retinal degeneration. The accompanying family data was used for confirmatory genetic interpretation. Retrospective chart data were reviewed, extending to the patients’ initial presentations at the study centre, which may have preceded their enrolment. Causative variants were identified in *CLN1* (*PPT1*), *CLN2* (*TPP1*), *CLN3*, *CLN5*, and *CLN7* (*MFSD8*), with three unrelated families harbouring *CLN7*-associated disease.

*CLN1-* patient presented with panuveitis and *CLN7*- were all Indian patients who had both anterior uveitis and intermediate uveitis (IU) which was seen during their follow-up of their genetic disease. *CLN5-* patient had IU. The *CLN2-* and *CLN3-* did not have any ocular inflammation.

All patients had adult-onset visual symptoms. Retinal phenotypes included rod–cone dystrophy in patients with *CLN1*-, *CLN2*-, *CLN3*-, and *CLN7*-associated disease, and cone–rod dystrophy with macular atrophy in one patient with *CLN5*-associated disease. Only one patient, with *CLN2*-associated disease, exhibited a possible syndromic manifestation in the form of adult-onset neurosensory hearing loss. The remaining patients had no neurological features at last follow-up.

Patient IRD0827 was found to harbour compound heterozygous variants in *CLN1* (*PPT1*): c.272 A > C (p.Gln91Pro; pathogenic by ACMG criteria), and c.491G > A (p.Arg164Gln; likely pathogenic by ACMG criteria) [16]. Parental testing confirmed the variants were *in trans*. The retinal phenotype was consistent with previously reported *CLN1*-associated retinal degeneration [16]. 

## Discussion

In this study, we detail a multi-ethnic Asian cohort presenting with adult-onset, predominantly non-syndromic CLN-associated retinal degeneration that originally masqueraded as uveitis or inflammatory retinopathy. Our central observation is that these patients frequently exhibit ocular inflammation, respond to standard immunosuppression, except our illustrative patient and suffer progressive retinal decline regardless of anti-inflammatory therapy. Crucially, we also demonstrate that even when active inflammation is absent, the clinical and structural imaging overlap with inflammatory disease easily misleads clinicians. This overlap is a major driver of diagnostic delay, underscoring exactly why clinicians must keep IRD—like CLN-associated disorders—high on the differential for any symmetrical, progressive posterior uveitis that fails to improve with conventional treatment.

Neurological investigations were not performed systematically in all patients as none of them had neurological symptoms and this may be a limitation of this cohort.

Uveitis masquerade syndromes remain a well-recognised diagnostic challenge, accounting for a small but clinically significant proportion of uveitis referrals. While neoplastic masquerades are often prioritised, non-neoplastic causes—including IRDs—are increasingly identified as important contributors. Previous series have demonstrated that IRDs represent up to one-third of non-neoplastic inflammatory masquerades and are associated with the longest delays to diagnosis. Our findings are consistent with these observations and extend them by identifying *CLN*-associated retinal degeneration as a specific and under-recognised cause of inflammatory masquerade in adults.

Neuronal ceroid lipofuscinoses encompass a clinically heterogeneous spectrum of lysosomal disorders that may present during infancy, late childhood, adolescence, or adulthood, with variable combinations of retinal degeneration and systemic neurological involvement [17, 18]. However, growing evidence indicates that certain *CLN* genotypes can present in adulthood with non-syndromic or predominantly ocular disease. In our cohort, all patients had adult-onset visual symptoms, and only one individual exhibited a possible extra-ocular manifestation. This reinforces emerging genotype–phenotype correlations in which adult-onset *CLN1*, *CLN2*, *CLN3*, *CLN5*, and *CLN7* variants may manifest as non-syndromic retinal dystrophies for prolonged periods. Although NCL classically present with severe neurological decline, atypical variants can manifest solely as isolated retinal degeneration [19, 20]. Systemic surveillance remains essential, as these phenotypes often mimic typical IRD before any systemic manifestations become apparent; however, some patients maintain a disease course restricted entirely to the eye. For instance, specific *CLN3* mutations have been shown to cause early- or late-onset rod-cone dystrophy with severe photoreceptor loss, completely sparing patients from the classic cognitive and psychomotor deterioration [19]. Furthermore, broader analyses highlight significant phenotypic variability across *CLN* genes, confirming that distinct pathogenic variants can produce strictly localized retinal disease and reinforcing the need for continuous longitudinal follow-up [20]. 

Inflammatory features were observed in most patients in this series, including anterior chamber cells, vitreous cells, retinal vasculitis without occlusion, optic disc hyperaemia, and CME. These findings overlap substantially with posterior uveitis and autoimmune retinopathy, contributing to misdiagnosis and prolonged immunosuppressive treatment as in our illustrative case. Importantly, immunosuppression resulted in minimal or transient improvement and did not alter the progressive course of retinal degeneration in our *CLN1-* patient. This pattern-symmetrical disease limited steroid responsiveness, and inexorable outer retinal loss—should prompt reconsideration of an inflammatory diagnosis and early genetic testing. Several IRD may present as uveitis masquerades and should be considered in the differential diagnosis.

### Key genes linked to uveitis in IRD

While NCL provides a well-recognised example of an IRD that can masquerade as uveitis, it is not unique in this regard. A growing number of IRD-associated genotypes have been linked to inflammatory or uveitis-like phenotypes that can obscure the underlying retinal degeneration. For example, pathogenic variants in PRPF8, PRPF31, and CRB1 have been associated with intermediate uveitis and systemic immune abnormalities, often leading to an initial impression of primary autoimmune disease rather than a genetic retinal disorder [21, 22]. Similarly, VCAN-related disease may present with marked vitreoretinal degeneration accompanied by inflammatory features that further complicate the diagnostic picture [23]. 

Certain genes—most notably *CAPN5*, *CRB1*, *ALPK1*, and *VCAN*—are now consistently linked to inflammatory ocular phenotypes [24]. We see this clinical overlap most strikingly in conditions like *CAPN5*-associated autosomal dominant neovascular inflammatory vitreoretinopathy (ADNIV) and *ALPK1*-related ROSAH syndrome. In these patients, recurrent uveitis, vitreoretinal fibrosis, and secondary neovascular complications often dominate the clinical picture, easily masking the underlying genetic cause [25–28]. Similarly, *CRB1* mutations remain the most frequent culprit among IRD presenting as non-infectious uveitis. Taken together, these patterns strongly suggest that the structural breakdown of the retina, coupled with blood-retinal barrier failure and localized immune dysregulation, actively drives the uveitis seen in these IRD [24, 29]. Table 3 shows shared features between IRD and uveitic entities [30–33]. 

**Table 3:** shows shared features of inherited retinal diseases and uveitis

Category	Shared Feature	Description & Evidence
Clinical Manifestations	Cystoid Macular Edema (CME)	A major cause of vision loss in both; in IRDs, it is a secondary complication of chronic inflammation.
	Vitreous Involvement	IRD patients often present with vitritis or “vitreous haze,” which is a primary hallmark of intermediate and posterior uveitis.
	Vascular Abnormalities	Both exhibit retinal vascular leakage, perivascular sheathing, and vascular staining on fluorescein angiography.
	Optic Nerve Changes	Optic disc hyperfluorescence and edema are common in both classic uveitis and specific IRDs like *EYS* or *ALPK1* mutations.
Biological Mechanisms	Innate Immune Activation	Both involve the activation of retinal microglia and Müller glia, which release pro-inflammatory cytokines like IL-6 and TNFα
	Adaptive Autoimmunity	Shedding photoreceptor discs in IRDs can trigger an autoimmune response similar to primary autoimmune uveitis.
	Barrier Dysfunction	Breakdown of the BRB and ELM is a core shared mechanism.
	Autoantibodies	High levels of AR-AAbs against antigens like S-antigen are prevalent in both populations.
Diagnostic & Systemic	Masquerade Effect	IRDs represent ~ 31% of non-neoplastic inflammatory masquerade syndromes, often leading to initial misdiagnosis as uveitis.
	Systemic Inflammation	Certain IRDs, such as ROSAH syndrome, mirror systemic uveitis by presenting with recurrent fever and splenomegaly.

CME- Cystoid macular edema, IRD- Inherited retinal diseases, IL-6- Interleukin 6, TNFα- Tumor necrosis factorα, BRB- Blood-retinal barrier, ELM- External limiting membrane, AR-AAbs -Anti-retinal autoantibodies

ROSAH syndrome is a rare, autosomal dominant autoinflammatory disorder. The name is an acronym for its primary clinical features: Retinal dystrophy, Optic nerve edema, Splenomegaly, Anhidrosis, and Headache

Awareness of this broader inflammatory spectrum is therefore important when evaluating patients with atypical or treatment-resistant uveitis, as early consideration of an IRD may prompt timely genetic testing and help avoid prolonged exposure to potentially ineffective immunosuppressive therapies.

Compared with these entities, *CLN*-associated retinal degeneration is distinguished by progressive pan-retinal dysfunction, characteristic autofluorescence abnormalities, and severe electrophysiological impairment, often in the absence of systemic features at presentation. Awareness of this phenotype may facilitate earlier diagnosis. Multimodal imaging and electrophysiological testing may be particularly useful in distinguishing IRD from inflammatory posterior uveitic entities. Bilateral symmetry of retinal degeneration, progressive ellipsoid zone loss, generalized outer retinal atrophy, characteristic autofluorescence abnormalities, constricting visual field defects, and diffuse rod-cone dysfunction on full-field ERG may collectively support a degenerative rather than primarily inflammatory processes. Recognition of these overlapping phenotypes is increasingly important within ocular inflammation practice, particularly in patients with bilateral symmetrical retinal degeneration and varied response to immunosuppressive therapy.

From a population perspective, *CLN*-associated retinal degeneration accounted for 1.1% of IRD families at our centre, indicating that these conditions, while rare, are not exceptional in a tertiary referral setting. Given increasing access to genetic testing and emerging gene- and enzyme-based therapies for *CLN* disorders, timely diagnosis has growing implications for patient counselling, surveillance for systemic involvement, and future therapeutic eligibility.

The illustrative case of an adult-onset, non-syndromic *CLN1*-associated retinal degeneration underscores the clinical consequences of delayed recognition. The patient underwent multiple systemic immunosuppressive therapies over couple of years before genetic testing established the diagnosis. Although inflammatory signs like macular oedema, initially thought to be CME did not leak on angiography. Closer look showed that these cystic spaces were a result of loss of retinal tissue may be managed symptomatically, the underlying degenerative process continued unabated. This case exemplifies how misclassification of inherited retinal degeneration as inflammatory disease can expose patients to unnecessary treatment-related risks without therapeutic benefit. There is a notable gap in our clinical research when it comes to uveitis specifically occurring within the landscape of IRDs. While current data suggests that certain immunomodulatory therapies can be highly effective—depending on how they engage with a disease’s specific biological pathways—the rarity of this clinical overlap means we are still operating with limited data. Most of what we know today comes from isolated case reports or small clinical series, rather than the robust, large-scale studies we would prefer.

When clinicians are faced with treating CME in the presence of an IRD, the “go-to” first-line therapy is typically a combination of oral and topical carbonic anhydrase inhibitors (CAIs) [33, 34]. This preference is driven by the fact that these medications specifically target the membrane-bound carbonic anhydrase II found within the retinal pigment epithelium (RPE) [35, 36]. 

However, standard CAI therapy does not work for everyone. For patients who remain non-responsive, several other medical and surgical avenues have shown success in reducing macular swelling in retinitis pigmentosa [34, 37]. These alternatives range from local or systemic corticosteroids (available in oral or intravitreal forms) to intravitreal anti-VEGF injections [38]. In some instances, patients may also find relief through topical non-steroidal anti-inflammatories like ketorolac, or through procedural options like grid laser photocoagulation and pars plana vitrectomy [33]. Tocilizumab has been reported in isolated refractory cases of CME; however, current evidence remains limited to anecdotal reports, and its efficacy and safety in IRD-associated inflammatory CME have not been established [39]. In this cohort *CLN1-* presented with severe inflammation, while *CLN7-* had AU and IU during the disease which responded to immunosuppression with steroids.

The limitations of this study include its retrospective design, small sample size, and referral bias inherent to a tertiary centre. Neurological investigations were not systematically performed in all patients, and longer follow-up is required to determine the risk of late-onset systemic manifestations. Nevertheless, this cohort represents one of the largest descriptions of adult-onset *CLN*-associated retinal degeneration presenting as inflammatory masquerade in an Asian population.

*CLN*-associated retinal degeneration, and indeed other forms of IRD, should always be considered in the differential diagnosis for patients with symmetrical posterior uveitis, progressive retinal degeneration, and variable response to immunosuppression. The other patients with confirmed diagnosis of IRD may also present with inflammation during their natural history and may cause diagnostic dilemma. Early genetic testing is critical to establishing the correct diagnosis, avoiding unnecessary long-term immunosuppressive therapy, and enabling appropriate counselling and surveillance. Recognition of this entity expands the differential diagnosis of uveitis masquerade syndromes and underscores the importance of integrating genetic evaluation into the assessment of atypical or treatment-resistant posterior uveitis.

## Data Availability

No datasets were generated or analysed during the current study.

## Abbreviations

ABCC6: ATP-binding cassette subfamily C member 6
ACMG: American College of Medical Genetics and Genomics
ALPK1: Alpha Kinase 1
AR-AAbs: Anti-retinal autoantibodies
AU: Anterior uveitis
BCVA: Best-corrected visual acuity
BRB: Blood-retinal barrier
CAI(s): Carbonic anhydrase inhibitor(s)
CRB1: Crumbs Cell Polarity Complex Component 1
CRD: Cone-rod dystrophy
CLN: Ceroid lipofuscinosis neuronal gene / CLN gene family
CME: Cystoid macular edema
DA: Dark-adapted
ELM: External limiting membrane
ERG: Electroretinogram
EZ: Ellipsoid zone
F: Female
FFA: Fundus fluorescein angiography
FEVR: Familial exudative vitreoretinopathy
IL-6: Interleukin 6
IMT: Immunomodulatory therapy / immunosuppressive therapy
IRD: Inherited retinal disease(s)
IU: Intermediate uveitis
LA: Light-adapted
logMAR: Logarithm of the minimum angle of resolution
M: Male
MD: Mean deviation
mfERG: Multifocal electroretinogram
NCL: Neuronal ceroid lipofuscinosis
OCT: Optical coherence tomography
OD: Right eye
OS: Left eye
PC: Periocular
PERG: Pattern electroretinogram
PRPF8: Pre-mRNA Processing Factor 8
PRPF31: Pre-mRNA Processing Factor 31
PPT1: Palmitoyl-protein thioesterase 1
PRISM: Precision Medicine Initiative
RCD: Rod-cone dystrophy
RPE: Retinal pigment epithelium
ROSAH: Retinal dystrophy, optic nerve edema, splenomegaly, anhidrosis, headache
SUN: Standardization of Uveitis Nomenclature
TCZ: Tocilizumab
TPP1: Tripeptidyl peptidase 1
TNF: Tumor necrosis factor
TB: Tuberculosis
VFI: Visual field index
VCAN: Versican
VUS: Variant of uncertain significance
WES: Whole-exome sequencing
